# Clinical outcomes and risk factors in the surgical treatment of distal femur fractures

**DOI:** 10.1007/s00068-026-03328-9

**Published:** 2026-09-15

**Authors:** Lina F. Höller, Sebastian Höller, Philine Wittur, Stephan Sehmisch, Wolfgang Lehmann, Lukas Weiser

**Affiliations:** 1 Department of Trauma Surgery, Orthopaedics and Plastic Surgery, University Medical Center Göttingen (UMG), 37099 Göttingen, Germany; 2 Department of Trauma Surgery, Medical School Hannover, 30625 Hannover, Germany

**Keywords:** Distal femoral fracture, Surgical treatment, Clinical outcomes, Risk factor

## Abstract

**Purpose:**

Distal femoral fractures are rare but severe injuries with high complication rates, particularly in elderly or multimorbid patients. This study aimed to analyze complication profiles and identify risk factors for surgery-related and non-surgery-related complications following operative treatment.

**Methods:**

A retrospective cohort study was conducted including all adult patients undergoing surgical treatment of distal femur fractures at a Level I trauma center between 2012 and 2018. Demographic, pre-operative, operative, and postoperative characteristics were analyzed. Risk factors were assessed using univariate statistics.

**Results:**

A total of 56 patients with 60 distal femur fractures were included. Surgery-related complications occurred in 11.7% of cases, while non-surgery-related complications were observed in 58.3%. In univariate analysis, gender (*p* = 0.004), age (*p* = 0.014), BMI (*p* = 0.006), osteoporosis (*p* = 0.047), open fracture (*p* < 0.001), high-energetic trauma (0.031) and time to surgery (*p* = 0.024) were significant risk factors for surgery-specific complications. For non-surgery-related complications, need of an external fixator for primary surgical care and type of operation were significant risk factors in univariate analysis. In multivariable logistic regression analysis, need of an external fixator was the only significant risk factor for developing non-surgery-related complications (OR 9.9; *p* = 0.005).

**Conclusion:**

Distal femoral fractures exhibit high complication rates. The need of external fixation was identified as independent statistically significant risk factor for non-surgery related complications (thromboembolic complications, pulmonary complications, urinary tract infection, anemia, etc.). Risk factors for surgery-specific complications could not be validated in multivariable analysis. Therefore, particular emphasis should be placed on thrombosis prevention and infection prevention in patients with an external fixator.

## Introduction

With a prevalence of 0.4% of all fractures and 3–6% of all femur fractures, distal femur fractures are a rather rare fracture entity in humans [1–4]. Despite this, distal femur fractures have comparably high one-year mortality rates as the proximal femur fracture and are therefore of major clinical importance [4–6].

Irrespective of their aetiology, distal femur fractures are often complex fractures with joint involvement, dislocated fragments or severe concomitant injuries [7]. With regard to the fracture mechanism, there is a bimodal distribution in connection with gender and age. While in the 1980s the largest proportion was formed by young men in combination with a high-energy trauma, the stronger peak now represents older women who have suffered a trivial fall, usually with pre-existing osteopenic bone structure [2, 8]. While the average age of men is under 50 years, the average age of women is significantly higher at over 70 years [9, 10]. Women are generally affected more often than men, with their proportion usually exceeding 60% [8, 10, 11].

The standard of care is surgical treatment which is considered technically demanding [12, 13]. Since the introduction of minimally invasive surgical procedures such as the distal femoral nail (DFN) or the locking compression plate (LCP), the previously remarkably high complication rate has fallen significantly [12]. However, even today, fracture treatment is still associated with numerous complications such as pseudarthrosis, infection or loosening of the material, which can lead to subsequent surgery or an unsatisfactory overall result [14].

Although several studies have investigated risk factors for complications following distal femur fracture fixation, the available evidence remains limited and heterogeneous. Patient-related factors such as increased body mass index, comorbidities, and smoking, as well as injury-related factors including open fractures and fracture severity, have been associated with non-union, infection, implant failure, or reoperation in individual studies [15–18]. However, the reported predictors are not consistent across cohorts. For example, while increased BMI and open fractures have been identified as independent risk factors for non-union, other studies have emphasized the relevance of surgical timing, soft-tissue injury, polytrauma, or fracture characteristics [15, 16]. Moreover, differences in study populations, fracture patterns, treatment strategies, definitions of complications, and indications for revision surgery make direct comparison between studies difficult [15–18]. Furthermore, most previous studies have focused predominantly on individual surgical complications, particularly non-union, infection, or implant failure, whereas potential predictors of broader postoperative morbidity have received less attention.

Due to the demographic change, the number of osteoporotic fractures is increasing, which will also include distal femur fractures [1, 19]. In addition, a 12% increase in the incidence of knee fractures has been demonstrated in a recent 20-year study [3]. Consequently, the prevalence of distal femur fractures is expected to increase, making them increasingly relevant, also in terms of the healthcare system and associated costs.

The aim of this study was therefore to identify patient-, injury-, and treatment-related factors associated with complications following operative treatment of distal femur fractures. Complications were analyzed separately as surgery-specific and non-surgery-related complications to account for the potentially different risk profiles underlying these outcomes. We hypothesized that patient and injury characteristics, as well as treatment-related factors, are associated with an increased risk of postoperative complications and that the predictors of surgery-specific complications differ from those of non-surgery-related complications.

## Methods

A retrospective cohort study was conducted including patients who underwent surgical treatment of distal femur fractures between 2012 and 2018 at the University Medical Center Göttingen. The local ethics committee (University Medical Center Göttingen) approved this study (study number 4/11/22). All the research was performed in accordance with the principles of the Declaration of Helsinki.

Patients were eligible for inclusion if they were aged older than 18 years and treated primarily at the University Medical Center Göttingen. Initially, the data of 171 patients with the acute diagnosis of “distal femur fracture” were identified. After individual review of the cases, patients were excluded due to age under 18, incorrect coding, revision operations or treated because of removal of osteosynthesis material. Fractures with severity grade AO 33-A1.1 and fractures that were treated conservatively also led to exclusion from the study. One patient committed suicide, six patients died because of their concomitant injuries on the day of the accident or during their hospital stay before a definitive osteosynthetic treatment could be performed and were also not included in the study. There was one pathological fracture among the initial study participants; due to a defect in our archive, another patient file was not accessible, which is why these two participants were also not included in the analysis. Of the initial 171 fractures, a total of 96 cases had to be excluded, leaving 75 fractures that could be included in the study.

Electronic medical record systems were used to identify patient demographics and comorbidities. Baseline characteristics (age, sex) and risk factors (comorbidities, medication, smoking, body mass index (kg/m^2^)) were recorded. In addition, pre-operative and operative characteristics (such as open or closed fracture, classification of the fracture, type of operation) and clinical outcomes (such as complications and re-interventions, length of hospital stay) were recorded. As clinical outcomes, length of hospital stay, complications, re-interventions and death during hospital stay were analysed. In addition, the correlation with different demographic, pre-operative and operative characteristics were analysed.

Standard methods of descriptive statistics were used. Categorical data are presented as percentages. The normality of continuous variables was tested using the Shapiro-Wilk test. The Shapiro-Wilk test revealed a significant deviation from the normal distribution for all continuous data, therefore continuous data are presented as median combined with interquartile range (IQR). Differences between groups are assessed using chi-square tests. To compare the medians of the continuous variables, Mann-Whitney-U-tests are used. Multivariable logistic regression analysis was performed to significant factors of the univariate analysis. P values < 0.05 were considered statistically significant. All statistical analyses were performed with SPSS Statistics 24.

## Results

A total of 56 patients with 60 distal femur fractures, aged between 21 and 97 years, were included in this study. On average, the median age of all patients was 56.5 years (IQR 34.5). Within the patient collective, 63.3% of all patients were female and 37.7% were male. With an average age of 44.5 years, men were significantly younger than women, with an average age of 69 years (*p* < 0.001). The average body mass index of the patients was 24.48 (IQR 5.55). The results of the analysis of additional comorbidities and risk factors are shown in Table 1.

**Table 1:** Patient demographics and comorbidities

	*n* = 60
**Age (years)**	56.5 (IQR 34.5)
**Gender**	
Female	38 (63.3%)
Male	22 (36.7%)
**Comorbidities**	
BMI (kg/m²)	24.48 (IQR 5.55)
Smoking	10 (16.7%)
Arterial hypertension	21 (35%)
Diabetes mellitus	7 (11.7%)
Obesity	7 (11.7%)
Dementia	8 (13.3%)
Osteoporosis	20 (33.3%)
**ASA Score**	
ASA 1	9 (15.7%)
ASA 2	38 (62.7%)
ASA 3	9 (15.7%)
ASA 4	4 (5.9%)

Data presented as median + interquartile range (IQR) (for continuous data) or percentage (for categorical data)

BMI = body mass index

In our cohort, AO type C fractures were the most common (51.7%). In total, 28.3% of the patients presented with an open fracture. Most common cause of the distal femur fracture was a low-energetic fall with 45%, followed by high-energy trauma in 43.3% of the patients. When the cause of fracture was correlated with gender and age, a bimodal distribution was evident. The largest peak was formed by women over 70 years of age who suffered a fall, the second peak consisted of men under 50 years of age who sustained the fracture due to a high-energetic trauma (both *p* < 0.05). With regard to the surgical care, an external fixator for primary care was fitted to 20 of the patients (33.3%). As final surgical care, most patients received a plate osteosynthesis (88.3%). All details regarding the operative characteristics are shown in Table 2.

**Table 2:** Pre-operative and operative characteristics

	*n* = 60
**AO classification**	
Type A	17 (28.3%)
Type B	12 (20%)
Type C	31 (51.7%)
**Soft tissue damage**	
Closed fracture	43 (71.7%)
Open fracture	17 (28.3%)
**Cause of fracture**	
Polytrauma / High energetic trauma	26 (43.3%)
Low energetic fall	27 (45%)
Other	7 (11.7%)
External fixator	20 (33.3%)
Time interval until first surgery (days)	1 (IQR 1) (1–60)
**Type of operation**	
Screw osteosynthesis	5 (8.3%)
Plate osteosynthesis	53 (88.3%)
Distal femoral replacement	1 (1.7%)
External fixator left in place	1 (1.7%)

Data presented as median + interquartile range (IQR) (for continuous data) or percentage (for categorical data)

### Clinical endpoints

In our study, 38% (23/60) were followed up over a period of at least 6 months, 30% (18/60) over at least one year and 15% (9/60) over two years. 26.7% were lost to follow-up. The average length of hospital stay was 16.5 days (IQR 27.25). The surgery-specific complication rate was 11.7%. Some patients presented with several complications combined. The most common specific complication was an infection in five patients. A re-intervention was needed in seven patients with complications (11.7%) and one patient died during hospital stay (1.7%). Most common non-surgery-related complications were anaemia and pneumonia with 50% and 20% respectively (Table 3).

**Table 3:** Clinical endpoints

	*n* = 60
Length of hospital stay (days)	16.5 (IQR 27.25) (range 5–82)
**Specific complications**	**11.7%**
Infection	5 (8.3%)
Pseudarthrosis	3 (5%)
Compartment syndrome	1 (1.7%)
Dislocation of the osteosynthesis material	1 (1.7%)
**Re-intervention**	7 (11.7%)
**Death during hospital stay**	1 (1.7%)
**Non-surgery-related complications**	**58.3%**
Anaemia	30 (50%)
Pneumonia	12 (20%)
Urinary tract infection	12 (20%)
Electrolyte imbalance	11 (18.3%)
Renal failure	6 (10%)
Cardiac complications	5 (8.3%)
Bacteremia	5 (8.3%)
Thromboembolic complications	3 (5%)

Data presented as median + interquartile range (IQR) (for continuous data) or percentage (for categorical data)

When comparing the association of gender with the development of complications, it became apparent that non-surgery-related complications were distributed equally over the overall collective, but specific complications occurred significantly more frequently in men (*p* = 0.004). A significant association between surgery-related complications and age was found as well (*p* = 0.014). Furthermore, a statistical association was found between specific complications and osteoporosis (*p* = 0.047) and BMI (*p* = 0.006).

No significant association was found between classification of the fracture and complications post-operatively. After grouping the causes of fracture into low-energetic fall, high-energetic trauma and other causes, a statistical association with specific complications (*p* = 0.031), but not with non-surgery-related complications (*p* = 0.069), was demonstrable. Furthermore, there was an association between the presence of osteoporosis and the fracture mechanism (*p* < 0.01). All patients with osteoporosis had a low-energetic fall as cause of the fracture (27/27). There was a statistical association between the time to surgical treatment and the complications (specific complications *p* = 0.024; non-surgery-related complications *p* = 0.474). Open fractures were identified as potential predictors for specific complications (*p* < 0.001). Patients who needed an external fixator for primary surgery care developed more non-surgery-related complications (*p* = 0.003). Another statistically significant association was found between non-surgery-related complications and type of operation (*p* = 0.02).

In conclusion, in univariate analysis, gender, age, BMI, osteoporosis, open fracture, high-energetic trauma and time to surgery were significant predictors for surgery-specific complications. A multivariable logistic regression analysis of these factors was unsuccessful because of insufficient statistical power. For non-surgery-related complications, need of an external fixator for primary surgical care and type of operation were significant predictors in univariate analysis. In multivariable logistic regression analysis with both factors, need of an external fixator was still a significant risk factor for developing non-surgery-related complications (OR 9.9; *p* = 0.005).

Patients with specific complications spent an average of ten days more (34 days vs. 16.5 days) in hospital than the overall patient collective. Looking at the length of hospitalization of patients who suffered a polytrauma, it is noticeable that the average length of stay was twice as long as well (34.5 days) compared to patients who did not suffer a polytrauma. Similar results were also seen in the length of hospital stay for fractures that required initial treatment with an external fixator. Fractures that required this temporary treatment also had a mean length of stay that was more than twice as long (39 days) compared to patients for whom the use of a fixator could be avoided. Here, the average length of stay was only 12 days.

## Discussion

Distal femur fractures are rather rare fractures compared to other type of fractures, but with high clinical importance due to high mortality rates [1–4]. The aim of this study was to provide an up-to-date overview of the epidemiology, surgical care, and outcome of distal femur fractures and to identify correlations and possible risk factors, which may enable us to make recommendations for therapy optimization and contribute to adequate patient education.

The average age of 56.5 years determined in our study and the higher percentage of women of 63.3% were in line with the results of recent studies [5, 9, 11, 20, 21]. Men had a mean age of 44.5 years, while women were significantly older with a mean age of 60 years (*p* < 0.01). One quarter of all patients were 80 years or older (25%), all of them were female. One third of the total collective showed an age of less than 50 years, with a male proportion of 70%. These results, with a large proportion of older women and a smaller peak of young men, reflected the expected, typical distribution of distal femur fractures described in different studies [8–10]. Also regarding the cause of fracture in correlation with gender, a typical distribution was seen in our study compared to the literature. Khan et al. demonstrated a statistically significant difference (*p* < 0.01) between the cause and gender in their study with almost 125 participants: High-energetic trauma was more common in men (70.5%), while low-energetic falls were more common in women (82.7%) [8]. Similar results were also found by Elsoe et al. in a study with over 300 participants [9]. In our study, women with low-energetic falls as cause of fracture also accounted for the majority at 78.6%, while this was the case for men in combination with high-energetic trauma at 61.5%.

Our results showed a surgery-specific complication rate of 11.7%. This is comparable with the literature. Campana et al. described in their retrospective study including 42 patients seven cases of complications [11]. Dang et al. even reported a re-operation rate of 36% in their study with 74 patients [21]. We identified male gender as potential predictor for surgery-specific complications. As most patients were female, this result was not expected. An association between gender and specific complications has not been investigated in other studies. In a large study with over 350 participants, Zhu et al. showed no correlation between gender and postoperative infections in distal femoral fractures (*p* = 0.056) [22]. Due to the small size of our sample, further studies with larger patient groups are required to verify our results.

Second, we identified BMI as potential predictor for surgery-specific complications. In a study by Weinlein et al., BMI was identified as an independent risk factor for systemic complications (*p* < 0.01) and increased mortality (*p* < 0.01) of femoral fractures [23]. Obesity, defined as a BMI ≥ 30 kg/m², is known to be an independent risk factor for pseudarthrosis and wound infection, and is considered a risk factor for poorer outcome of distal femoral fractures compared to patients without obesity [24–26]. A study by Bryant et al. with over 330 patients showed that obesity was associated with an increased risk of prolonged hospitalization for femoral fractures, and the incidence of complications was twice as high as in normal weight patients [24]. Since obese patients in our study spent more twice as long in hospital than normal-weight patients, we confirmed this statement (29 vs. 15 days). Bryant et al. attributed the poorer outcome and the increased systemic complication rate to delayed mobilization and demonstrated a significant indirect effect in this regard (*p* < 0.05). Therefore, an association between the outcome or complication rate and an increased BMI was expected.

An important finding of this study is that patient-related factors such as BMI, osteoporosis, and comorbidities appear to have a stronger association with complications than fracture characteristics themselves. This underlines the importance of a patient-centered approach in perioperative risk assessment.

As last comorbidity, we identified osteoporosis as risk factor for surgery-related complications. Osteoporosis is associated with reduced bone mass and inferior bone substance, and both factors may lead to more difficult anchoring of the osteosynthesis material in the bone. In addition, osteoporosis leads to impaired and delayed fracture healing and thus predisposes to mechanical complications such as dislocation of the osteosynthesis material [27, 28]. Osteoporosis is also known to be a risk factor for pseudarthrosis, which was also counted among the specific complications in our study [29]. Contrary to our initial assumption, no statistical association between the presence of osteoporosis and fracture classification, i.e. fracture severity, could be demonstrated. However, an association was found between specific-complication and fracture mechanism (*p* = 0.031). All patients with known osteoporosis had a low-energetic fall as cause of fracture. This seems plausible, as low-energetic falls are considered one of the most common causes of fragility fractures and osteoporosis is associated with an increased risk of fracture even with minor force [30].

Regarding the operative characteristics, need of an external fixator for primary surgery care was identified as risk factor for non-surgery-related complications. Even in multivariable analysis, the risk for developing non-surgery-related complications was much higher in patients with external fixator (OR 9.9; *p* = 0.005) Due to immobilization and longer hospital stay patients are more at risk for development of complications such as thromboembolic events, nosocomial pneumoniae or urinary tract infections. Although patients after high-energetic trauma suffering a polytrauma had a longer hospital stay of more than 15 days as well, which is considered a risk factor for postoperative complications, the presence of a polytrauma was only proven to be a risk factor for specific complications in our study [31]. This can possibly be explained by the fact that patients with polytrauma were younger and had fewer previous comorbidities than the overall collective and can therefore be assumed to be in a better physical condition to counteract non-surgery-related complications. Increased age has been shown to be a risk factor for postoperative complications such as nosocomial pneumonia, urinary tract infections or thromboembolic events [32–35].

Furthermore, open fractures were identified as potential predictor for surgery-related complications. Initial soft tissue damage as a risk factor for complications in distal femoral fractures have generally only been investigated in a few studies and have not been investigated in relation to a group of summarized complications such as ours. However, the finding is supported by retrospective studies that have shown an association between open fractures and infection and between open fractures and pseudarthrosis in distal femur fractures [16, 18, 25, 36–38]. Higher rates of infection and/or pseudarthrosis are related to open fractures.

### Limitations

When interpreting and discussing the results presented, the following limitations of the study conducted should be considered. Most parts of this study have a purely retrospective character. Due to this retrospective design, recall bias and missing data may have influenced the outcomes. Furthermore, a bias in the evaluation and interpretation of the data collected cannot be ruled out. Even though our case number of *n* = 60 is relatively large for such a rare fracture compared to other studies on distal femur fractures, it’s still a rather small number of patient cases for a study. However, due to the rarity of this fracture, it is difficult to achieve a patient cohort large enough to exclude or greatly minimize the probability of random variables. The results obtained should be evaluated in larger studies to achieve greater significance. It should also be noted that it was not possible to evaluate all patients up to two years postoperatively, as many follow-up examinations were not carried out at the University Medical Center Göttingen. Follow-up treatments that took place outside our clinic were only recorded in exceptional cases and could therefore not be evaluated by us. Furthermore, the low follow-up rate may have led to an underestimation of late complications such as non-union or implant failure.

We aimed to perform multivariate analysis to identify independent risk factors, but this was not possible for surgery specific complications. Due to the small number of specific complications (*n* = 7) relative to the number of risk factors examined (*n* = 7), the multivariate logistic regression model proved to be unstable. The resulting regression coefficients, odds ratios, and confidence intervals could therefore not be reliably interpreted. Accordingly, no further interpretation of the model was performed. Potential confounding factors remain undetected.

## Conclusion

Distal femoral fractures exhibit high complication rates. The need of external fixation was identified as independent statistically significant risk factor for non-surgery related complications (thromboembolic complications, pulmonary complications, urinary tract infection, anemia, etc.). Gender, age, BMI, osteoporosis, open fracture, high-energetic trauma and time to surgery were associated with an increased risk of surgery-specific complications but could not be validated in multivariable analysis. Although all these factors are not directly modifiable, awareness of these risk factors may help to optimize perioperative management and improve patient counseling. Particular emphasis should be placed on thrombosis prevention and infection prevention in patients with an external fixator.

## Data Availability

Data are available on request.
